# P-1448. Targeting Smoking Among People with HIV (PWH): Does Intensive Group Therapy Reduce Mortality?

**DOI:** 10.1093/ofid/ofae631.1620

**Published:** 2025-01-29

**Authors:** Rohan Goyal, Abner A Kahan, Jonathan Shuter

**Affiliations:** Montefiore Medical Center, Melville, New York; Albert Einstein College of Medecine, Brooklyn, New York; Albert Einstein College of Medicine, Bronx, New York

## Abstract

**Background:**

PWH in the United States smoke cigarettes at rates 2-3 times higher than the general population. Between 2014 and 2017 we conducted a randomized controlled trial (RCT) comparing Positively Smoke Free (PSF), an 8-session intensive group therapy intervention targeting HIV+ smokers, to brief advice to quit. Findings suggested that PSF conditioned participants had higher quit rates at both early and late (approximately 3-year) follow-up. In the present study, we sought to determine whether PSF conditioned participants had a significant difference in mortality compared to controls 7-10 years after study enrollment.

**Table 1.**
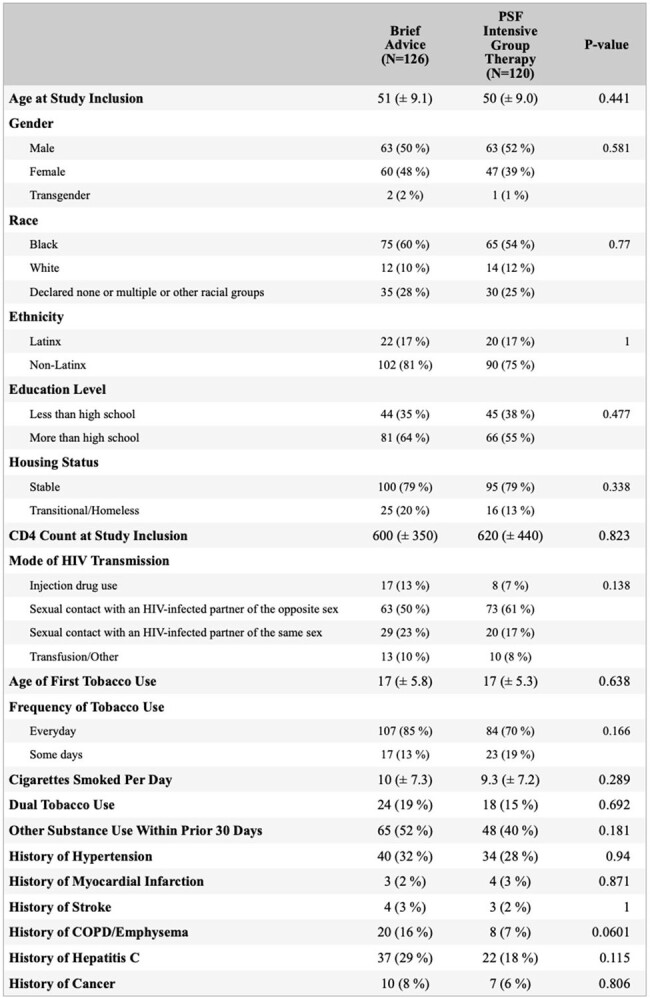
Baseline Characteristics of Study Participants.

**Methods:**

Participants of the Bronx cohort of the original RCT (n=247) were chart reviewed for vital status. Participants were classified as “alive” if review indicated contact with the patient within six months of review. If no records were available within the prior six months, an attempt was made to contact the patient, pharmacy, or emergency contact as listed either in the medical chart or original study records. Dates of death were recorded for deceased participants. Data were analyzed using R 4.4.0. All identifying patient information was secured in a password-protected spreadsheet only accessible to study authors.

**Figure 1.**
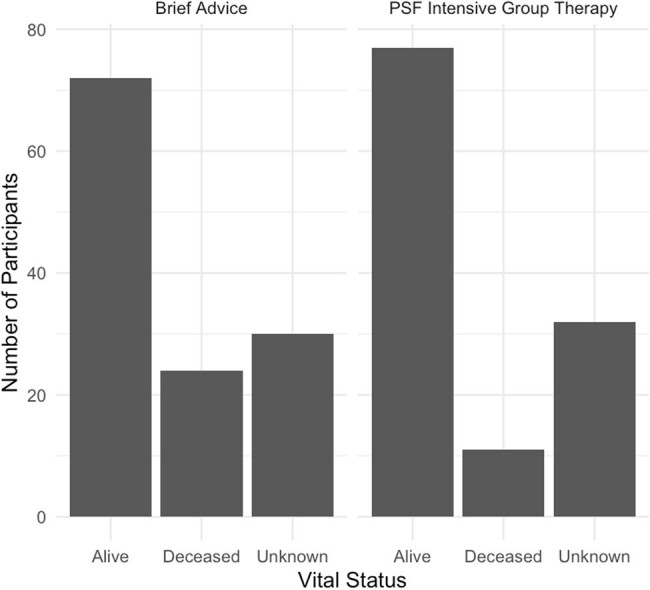
Vital Status of Study Participants.

**Results:**

There were no significant differences in baseline characteristics between treatment and control groups (Table 1). 14.2% of all participants were confirmed deceased at the time of data collection; vital status of 25.5% of participants could not be determined (Figure 1). Kaplan-Meier analysis was performed (Figure 2); log-rank test was significant for survival difference favoring the PSF vs control group (p = 0.03). Univariate Cox-proportional analysis showed a hazards ratio of 0.46 (0.23 - 0.94, p = 0.034).

**Figure 2.**
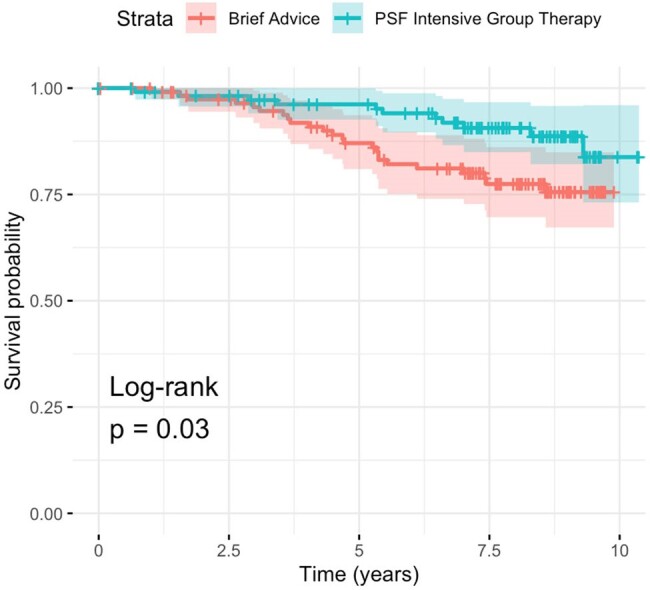
Kaplan-Meier Survival Analysis.

**Conclusion:**

Follow-up analysis of Bronx participants in the original RCT suggests that there is a significant mortality benefit over 7-10 years for HIV+ smokers who received PSF intensive group therapy.

**Disclosures:**

**All Authors**: No reported disclosures

